# Why Most Published Research Findings Are False: Author's Reply to Goodman and Greenland

**DOI:** 10.1371/journal.pmed.0040215

**Published:** 2007-06-26

**Authors:** John P. A Ioannidis

I thank Goodman and Greenland for their interesting comments [1] on my article [2]. Our methods and results are practically identical. However, some of my arguments are misrepresented:

1. I did not “claim that no study or combination of studies can ever provide convincing evidence.” In the illustrative examples (Table 4), there is a wide credibility gradient (0.1% to 85%) for different research designs and settings.
2. I did not assume that all significant *p*-values are around 0.05. Tables 1–3 and the respective positive predictive value (PPV) equations can use any *p*-value (alpha). Nevertheless, the *p* = 0.05 threshold is unfortunately entrenched in many scientific fields. Almost half of the “positive” findings in recent observational studies have *p*-values of 0.01–0.05 [3,4]; most “positive” trials and meta-analyses also have modest *p*-values.
3. I provided equations for calculating the credibility of research findings with or without bias. Even without any bias, PPV probably remains below 0.50 for most non-randomized, non-large-scale circumstances. Large trials and meta-analyses represent a minority of the literature.
4. Figure 1 shows that bias can indeed make a difference. The proposed modeling has an additional useful feature: As type I and II errors decrease, PPV(max) = 1 - [u/(R + u)], meaning that to allow a research finding to become more than 50% credible, we must first reduce bias at least below the pre-study odds of truth (u less than R). Numerous studies demonstrate the strong presence of bias across research designs: indicative reference lists appear in [5–7]. We should understand bias and minimize it, not ignore it.
5. “Hot fields”: Table 3 and Figure 2 present “the probability that at least one study, among several done on the same question, claims a statistically significant research finding.” They are not erroneous. Fields with many furtive competing teams may espouse significance-chasing behaviors, selectively highlighting “positive” results. Conversely, having many teams with transparent availability of all results and integration of data across teams leads to genuine progress. We need replication, not just discovery [5].
6. The claim by two leading Bayesian methodologists that a Bayesian approach is somewhat circular and questionable contradicts Greenland's own writings: “One misconception (of many) about Bayesian analyses is that prior distributions introduce assumptions that are more questionable than assumptions made by frequentist methods” [8].
7. Empirical data on the refutation rates for various research designs agree with the estimates obtained in the proposed modeling [9], not with estimates ignoring bias. Additional empirical research on these fronts would be very useful.

Scientific investigation is the noblest pursuit. I think we can improve the respect of the public for researchers by showing how difficult success is. Confidence in the research enterprise is probably undermined primarily when we claim that discoveries are more certain than they really are, and then the public, scientists, and patients suffer the painful refutations.
